# Fixation in Form-Acetic allows hyaluronic acid detection in mouse ovaries

**DOI:** 10.1530/RAF-21-0085

**Published:** 2021-12-10

**Authors:** R Appeltant, B V Adeniran, S A Williams

**Affiliations:** 1 Nuffield Department of Women’s and Reproductive Health, Women’s Centre, John Radcliffe Hospital, University of Oxford, Oxford, UK

## Abstract

To visualise tissues to determine the presence of disease or simply to understand anatomy, it is important to preserve fresh tissue. Fixatives are chemical solutions that preserve tissues to enable microscopic evaluation. However, some fixatives introduce artefact such as shrinkage of cells. Recently, a new fixative, Form-Acetic, was developed that is superior for preserving the structure of ovary tissue and allows investigation of ovary composition. One component of the ovary is hyaluronic acid (HA), which plays a crucial role in normal ovary function and fertility. Importantly, HA is sensitive to different fixative solutions. Therefore, it is meaningful to verify whether Form-Acetic is suitable for detecting HA. In this study, adult mouse ovaries were fixed in Form-Acetic and HA was detected. All HA-containing structures in the ovary were clearly distinguished which proves that the novel fixative allows the detection of HA.

Fixative-induced morphological alterations such as shrinkage can lead to misinterpretation of data. This phenomenon is especially noticed after fixation with 10% (v/v) neutral buffered formalin (NBF). Bouin’s solution reduces the shrinkage artefact but is not ideal for immuno-labelling because of its coagulative properties. In 2021, we developed a new fixative, Form-Acetic (Adeniran *et al*. 2021), which is composed of NBF supplemented with 5% acetic acid and allows exquisite preservation of the tissue while enabling a range of histological molecular analyses (Adeniran *et al*. 2021).

Hyaluronic acid (HA) is an indispensable component of the extracellular matrix in several vertebrate tissues and is the major component of the cumulus matrix, which plays a key role in* in vivo* fertilization (Salustri *et al*. 2004). In the ovary, HA is to be found not only in the cumulus matrix (Lo *et al*. 2019) but also in the theca cells and follicular fluid (Christensen *et al*. 2015, Rowley *et al*. 2020). Since HA is non-immunogenic, immunohistochemistry cannot be performed; instead, a HA binding protein (HABP) is routinely used (Rowley *et al.* 2020). It has recently been revealed that the detection of HA is influenced by the choice of fixative with alcohol-based fixatives (e.g. Carnoy’s solution) preferred (Rowley *et al.* 2020). However, although aldehyde-based fixatives (e.g. NBF) have been used to detect HA (Lo *et al.* 2019), detection of HA was not assessed after fixation with Form-Acetic (Adeniran *et al.* 2021). Consequently, it was important to verify whether Form-Acetic facilitated the detection of HA in the ovary. Duration of fixation can also affect the detection of molecules, and therefore, we compared 8 and 24 h fixation of mouse ovaries in Bouin’s, NBF and Form-Acetic.

Ovaries from 10 weeks old C57Bl/6 mice were fixed, paraffin-embedded and sectioned. For each condition, three different mice were used. Sections were subjected to a routine histochemistry protocol. In summary, endogenous peroxidase was blocked with 3% hydrogen peroxide in PBS. Non-specific binding sites were blocked using 2% fetal calf serum in PBS. Slides were incubated in biotinylated HABP (Calbiochem, Sigma Aldrich 385911) and the HA signal was detected using a Vectastain ABC Elite kit with DAB peroxidase substrate kit (Vector Laboratories). Within an experiment, the exposure time was either the ‘same duration’ of exposure (4 min 20 s) or exposed according to signal intensity (’variable’). Slides were dehydrated, mounted and images captured (Lumenera Infinity 5 camera using Infinity Analyze Software).

HA was clearly detected in follicular fluid, theca cell layers and between granulosa cells in Bouin’s, NBF and Form-Acetic irrespective of the duration of fixation (Fig. 1). When the same duration DAB exposure was used, the HA detected in NBF and Form-Acetic appears less than Bouin’s, but all the HA-containing structures were still clearly visible at both fixation times.

**Figure 1:**
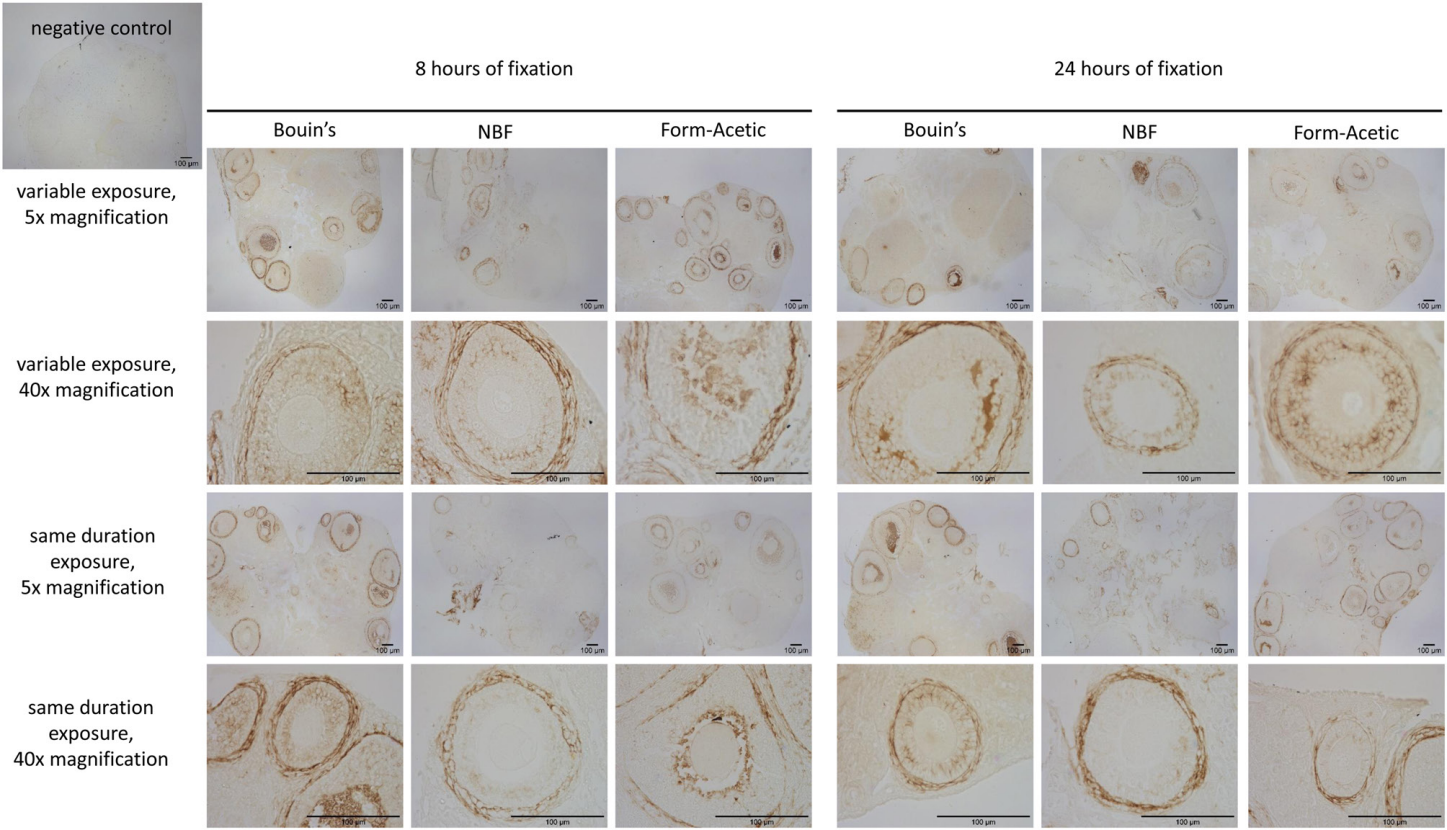
Detection of hyaluronic acid in mouse ovaries preserved in three fixatives (Bouin’s, 10% (v/v) NBF or Form-Acetic) after 8 or 24 h of fixation. A variable exposure refers to a different DAB exposure for each condition; a same duration exposure time refers to a common DAB incubation time (4 min 20 s) for all the conditions. NBF, neutral buffered formalin.

In conclusion, we can state that fixation of ovarian mouse tissue in the novel fixative Form-Acetic allows the detection of HA regardless of the fixation time.

## Declaration of interest

Suzannah Williams is an Associate Editor of *Reproduction and Fertility*. Suzannah Williams was not involved in the review or editorial process for this paper, on which she is listed as an author. There is no conflict of interest that could be perceived as prejudicing the impartiality of the research reported.

## Funding

This work was supported by the Rhino Fertility Project: Fondation Hoffmann.

## Author contribution statement

R Appeltant and S A Williams were involved in the conception and design of the study, data interpretation and manuscript generation. R Appeltant and B V Adeniran were both involved in data acquisition, with R Appeltant generating the bulk of the data, performing the data analysis and drafting the article. All authors contributed to and agreed the final draft of the manuscript.
